# Cortical reorganization in recent-onset tinnitus patients by the Heidelberg Model of Music Therapy

**DOI:** 10.3389/fnins.2015.00049

**Published:** 2015-02-19

**Authors:** Christoph M. Krick, Miriam Grapp, Jonas Daneshvar-Talebi, Wolfgang Reith, Peter K. Plinkert, Hans Volker Bolay

**Affiliations:** ^1^Department for Neuroradiology, Saarland University HospitalHomburg, Germany; ^2^German Center for Music Therapy Research (Victor Dulger Institute) DZMHeidelberg, Germany; ^3^Department of Otorhinolaryngology, Head and Neck Surgery, University Hospital for Ear, Nose, and Throat, University of HeidelbergHeidelberg, Germany; ^4^Music Therapy Tinnitus Outpatient Department, German Center for Music Therapy Research (Victor Dulger Institute) DZMHeidelberg, Germany

**Keywords:** tinnitus, cerebral reorganization, brain plasticity, auditory cortex, MRI, voxel-based morphometry (VBM), Heidelberg Model of Music Therapy, gray matter

## Abstract

Pathophysiology and treatment of tinnitus still are fields of intensive research. The neuroscientifically motivated Heidelberg Model of Music Therapy, previously developed by the German Center for Music Therapy Research, Heidelberg, Germany, was applied to explore its effects on individual distress and on brain structures. This therapy is a compact and fast application of nine consecutive 50-min sessions of individualized therapy implemented over 1 week. Clinical improvement and long-term effects over several years have previously been published. However, the underlying neural basis of the therapy's success has not yet been explored. In the current study, the therapy was applied to acute tinnitus patients (TG) and healthy active controls (AC). Non-treated patients were also included as passive controls (PTC). As predicted, the therapeutic intervention led to a significant decrease of tinnitus-related distress in TG compared to PTC. Before and after the study week, high-resolution MRT scans were obtained for each subject. Assessment by repeated measures design for several groups (Two-Way ANOVA) revealed structural gray matter (GM) increase in TG compared to PTC, comprising clusters in precuneus, medial superior frontal areas, and in the auditory cortex. This pattern was further applied as mask for general GM changes as induced by the therapy week. The therapy-like procedure in AC also elicited similar GM increases in precuneus and frontal regions. Comparison between structural effects in TG vs. AC was calculated within the mask for general GM changes to obtain specific effects in tinnitus patients, yielding GM increase in right Heschl's gyrus, right Rolandic operculum, and medial superior frontal regions. In line with recent findings on the crucial role of the auditory cortex in maintaining tinnitus-related distress, a causative relation between the therapy-related GM alterations in auditory areas and the long-lasting therapy effects can be assumed.

## Introduction

Tinnitus is one of the most common symptoms in ENT medicine (Pilgramm et al., 1999). Whereas short and transient phantom noise seems to be a ubiquitous phenomenon in general population, 5–15% of people are affected by a persisting manifestation, among those, up to 2% of cases being even severely restricted in their quality of life (Axelsson and Ringdahl, 1989; Khedr et al., 2010; Shargorodsky et al., 2010). Beyond that, the phantom noise often carries additional psychiatric, psychosocial, or psychosomatic comorbidities such as anxiety and depression, concentration and attention deficits, as well as sleep disorders (Jacques et al., 2013). This epidemiological profile points to the value of investigating the origins of tinnitus more thoroughly in order to get better understanding of its nature and of the potential for remediation. Tinnitus is thought to be triggered in many cases by cochlear damage, resulting in abnormal or missing afferent input to the auditory cortex (Moller, 2007). However, this specific defect seems not to be sufficient to explain the whole genesis. To date, there are many methods available to explore brain involvement in phantom noise (Galazyuk et al., 2012; Langguth et al., 2013; Noreña and Farley, 2013; Zhang, 2013) including, Transcranial Magnet Stimulation (Theodoroff and Folmer, 2013), Independent Component Analysis of brain potentials (De Ridder et al., 2011b) and morphometric measurements (Schecklmann et al., 2013). Recent neuroimaging studies of tinnitus indicate the involvement of wide-spread brain networks for perception, attention, memory, and emotional aversive processes (Adjamian et al., 2009; Lanting et al., 2009). In this context De Ridder et al. (2013) proposed a neuronal model of phantom perception and its emotional coupling to distress based on a previous model proposed by Jastreboff (1990). According to the authors, missing signals by sensory deafferentation cause high-frequency, gamma band, synchronized neuronal activity in the sensory cortex. This activity only reaches awareness when it co-activates brain networks that are related to self-perception and salience (amygdala, anterior cingulated, anterior insula, and precuneus). In such way getting conscious, the phantom percept also activates a non-specific distress network that in turn overlaps with salience coding, resulting in an emotional coupling of tinnitus to the experience of distress. Structural brain analysis observing neuronal plasticity has been found to be suitable for understanding correlations between such mental sensations and neural mechanisms (Valkanova et al., 2014). Of critical importance are seven studies so far that specifically investigated anatomical deviations in tinnitus (Mühlau et al., 2006; Landgrebe et al., 2009; Schneider et al., 2009; Husain et al., 2011; Leaver et al., 2012; Boyen et al., 2013; Schecklmann et al., 2013). Noteworthy, even though they all consistently report structural differences, their results largely differ regarding both the localization and the direction of changes. However, there is growing consensus on the involvement of gray matter (GM) alterations in auditory brain areas when suffering from tinnitus distress. For instance, Schecklmann et al. (2013) found such an interrelation in the course of a large cross-sectional morphometric study (*n* = 257), and cross-validated the results in an independent second sample (*n* = 78). More precisely, tinnitus distress correlated negatively with GM volume in bilateral auditory areas, pointing to higher individual tinnitus distress with lower gray matter volume. Similarly, Schneider et al. (2009) found gray matter loss associated with tinnitus in the Heschl's gyri, again indicating a close relationship between tinnitus and auditory cortices. Leaver et al. (2012) also revealed reduced gray matter next to auditory area in tinnitus patients, and additionally observed a substantial GM decrease in medial frontal cortex (dmPFC). The authors argued that the latter tinnitus-related alterations in dmPFC might not be related to distress, but to individual loudness of tinnitus sensation. Boyen et al. (2013) also observed changes in auditory areas due to tinnitus, but in contrast to the previous findings pointing to an increase of GM.

Suffering from tinnitus does not necessarily mean feeling diseased due to the phantom noise. Quite the contrary, neither perceived loudness nor tinnitus frequency seem to correlate with mental strain, but it is the emotional correlate of tinnitus, that is, tinnitus distress, which may trigger such feelings of diminished well-being (De Ridder et al., 2011a). In line with this assumption, GM alterations in auditory cortex were not correlated with tinnitus sound, but with severity of tinnitus-related distress (Schecklmann et al., 2013). Hence the existence of tinnitus *per se* (by phantom noise) does not require any therapeutic intervention. But since tinnitus often co-occurs with considerable emotional decline among affected patients, there is still demand for therapeutic assistance. However, many available therapies resulted in relatively small effects or lacked improvement in tinnitus load (Pichora-Fuller et al., 2013).

In case of acute tinnitus manifestation, existing treatment options may be considered unsatisfactory. On the one hand, several pharmacological approaches (Patterson and Balough, 2006) have been established considering tinnitus to be equivalent to sudden sensorineural hearing loss (Hesse and Laubert, 2010) or to any cochlear damage (Shim et al., 2011). However, none of these treatment methods have proven to be effective after replication in controlled trials (Elgoyhen and Langguth, 2011). On the other hand, different types of psychotherapeutic intervention supporting and accompanying medical treatment have also been designed (Schildt et al., 2006; Gerhards and Brehmer, 2010). These adjuvant psychotherapeutic interventions consist of one or more of the following elements: psycho-educative counseling, relaxation training, and general and tinnitus-related stress management. Different approaches designed to manage or to habituate the phantom noise have been established (Tinnitus Retraining Therapy, Cognitive Behavioral Therapy, Progressive Tinnitus Management, Biofeedback, Education, and Relaxation Therapies), partially resulting in persistent therapy success (Herraiz et al., 2007; Hesser et al., 2011; Folmer et al., 2014; Grewal et al., 2014; Myers et al., 2014). Whereas psychological strategies are intended to modulate attention and emotion toward tinnitus, noise maskers and hearing aids instead interact with acoustic sensation to suppress tinnitus perception. Tinnitus sound masking was developed in the early 1970s (Coles et al., 1984) and is still being used in mild cases, because a lasting improvement can be achieved as long as the external noise is applied. The devices led to reduced tinnitus distress, especially when combined with hearing aids amplifying the impaired frequency range (Oz et al., 2013). Direct modulating of the tinnitus-related activity is intended in either Transcranial Direct Current Stimulation (tDCS) or rapid Transcranial Magnet Stimulation (rTMS), too (Langguth and De Ridder, 2013). In most of these studies the primary auditory cortex has been targeted for tinnitus treatment by cortical stimulation (Simon et al., 2012). However, benefits from rTMS therapy have not been shown to persist over time (Theodoroff and Folmer, 2013).

Any effective therapy for tinnitus requires a fundamental understanding of its physiological and neural background. For instance, the “Heidelberg Model of music therapy for Tinnitus” refers to scientific evidence for cerebral circuits of tinnitus enhancement (Argstatter et al., 2008) (for details see Procedure section). This treatment approach strives for an integration of strategies to manage the psychological state and to possibly reverse the underlying neuronal reorganization. For this purpose, complementary music- and psychotherapeutic interventions, comprising emotional regulation of tinnitus load and exercises of frequency discrimination in the spectral range of tinnitus noise, have been organized into several modules, resulting in a manualized short-term music therapeutic treatment concept whose separate treatment modules and long term effects are described in detail by Argstatter et al. (2012). The authors also reported the high clinical efficacy and long-term effects of this approach in chronic tinnitus patients. Corresponding clinical therapeutic effects in patients with acute tinnitus have been previously reported by Grapp et al. (2013). The authors of this study measured a decrease of tinnitus-related mental load in treated compared to untreated patients after 1 week of therapy. This improvement on tinnitus distress by the aforementioned therapy concept formed the starting point of our research. We aimed to investigate the corresponding neural correlates of this distress-related improvement in tinnitus patients more thoroughly. Thus, we sought to gain a deeper insight into the complex brain etiology and into the possibility of cortical reorganization in tinnitus.

### Hypothesis

Based on previous studies on structural plasticity, we expected a neural correlate of the therapy effect to be most prominent within auditory areas (Heschl's gyri), as tinnitus distress is highly related to structural GM loss in these regions. Whereas micro-structural regeneration processes on a cellular level (Kwok et al., 2011) cannot be directly detected by MRI, corresponding effects on brain tissue (Kleim et al., 2004) seem to be reliably detectable by Voxel Based Morphometry (VBM) (Ashburner and Friston, 2000). By conflating specific evidence for structural changes after therapy (Seminowicz et al., 2013) with assumptions about the rapid intervention-induced expansion of GM as general principle of human neural plasticity (Driemeyer et al., 2008; Taubert et al., 2010; Tavor et al., 2013), we hypothesized a GM alteration also with the Heidelberg Model of Music Therapy after a short-term treatment interval of 1 week.

## Methods and participants

### Participants

In this study, we included participants who were diagnosed with acute tinnitus persisting for a maximum of 3 months, without significant symptom change after an initial medical intervention according to AWMF guidelines (glucocorticoids or rheological drugs). Before including the participants in music therapy, a waiting period up to 4 weeks was warranted in order to prevent both delayed drug response and the influence of possible spontaneous remission. After completion of this pharmacological treatment during the first weeks after tinnitus onset, tinnitus patients underwent a pre-participation evaluation for participation in the music therapy. In addition to standard audiological testing and otolaryngological examination, important demographic and tinnitus-related data were collected. Patients were excluded if the tinnitus was related to anatomic lesions of the ear, to retrocochlear lesions or to cochlear implantation. Further exclusion criteria comprised clinical diagnosis of a co-morbid severe mental disorder, clinical diagnosis of Menière's Disease, severe hyperacusis or severe hearing impairment more than 40 dB beyond the affected tinnitus frequencies. The latter criterion was chosen to exclude interaction between music therapy and hearing aids for the present.

Fifty patients with experience of a recent tinnitus onset (between 6 and 12 weeks prior to the intervention) were invited to participate in the music therapy study subsequent to treatment according to the standard clinical protocol for acute tinnitus in the University Hospital for Ear, Nose, and Throat at the University of Heidelberg. All patients had an age-appropriate hearing level and reported no otological or psychological co-morbidity. At the time point of the pre-participation evaluation (T_0_) the patients were randomly divided into two groups, a treatment group (TG) and a waiting group for passive tinnitus controls (PTC). The time span between tinnitus onset and T_0_ was 5.10 (SD 2.14) weeks in TG and 4.63 (SD 2.01) weeks in PTC. For ethical reasons, PTC patients also received the therapeutic intervention, but following the study period. Participants of both groups were instructed about MRI measurements and its noise level. All participants were insured for any health impairment and accidents. They gave written informed consent in accordance with the Declaration of Helsinki. The study was in accordance with the requirements of the ethic review board of Saarland.

After the period of the standard clinical treatment protocol, 7 patients were excluded from music therapy due to disappearance of tinnitus. Two further patients were excluded because of claustrophobia. Thus, the effective sample comprised 19 patients in the TG and 22 patients in the PTC. The patient groups did not differ in age, sex or in level of distress (see Table 1). The mean delay between tinnitus onset and therapy start (T_1_) was 8.14 (SD 1.85) weeks in TG and 8.10 (SD 1.45) weeks in PTC.

**Table 1:** **Patient-related as well as tinnitus-related data in an overview**.

	**TG** (***n*** = **20**)	**PTC** (***n*** = **22**)	**Statistics**
Tinnitus causation [acute hearing loss/noise trauma/distress/other] (n)	1/8/6/5	2/7/8/5	χ^2^(*df* = 1) = 0.489, *p* = 0.484
Type of tinnitus [tonal/non-tonal] (n)	11/9	12/10	χ^2^(*df* = 1) = 0.170, *p* = 0.680
Tinnitus frequency (Hz) [mean (SD)]	5102 (2332)	6376 (3176)	t(*df* = 41) = −1.469, *p* = 0.154
Tinnitus localization [right/left/bilateral/not determinable] (n)	5/7/5/3	4/9/5/4	χ^2^(*df* = 1) = 0.146, *p* = 0.703
TQ score from initial anamnestic diagnostics mean (SD)]	38.50 (15.4)	36.20 (16.82)	t(*df* = 41) = 0.737, *p* = 0.465
Tinnitus duration up to initial anamnestic diagnostics (T_0_)(weeks) [mean (SD)]	5.10 (2.14)	4.63 (2.01)	t(*df* = 41) = 0.567, *p* = 0.575
Tinnitus duration up to start of therapy (T_1_) (weeks) [mean (SD)]	8.14 (1.85)	8.10 (1.45)	t(*df* = 41) = 0.082, *p* = 0.935
Patients' age (years) [mean (SD)]	43.9 (10.4)	42.6 (11.5)	T(*df* = 43) = 0.31, *p* = 0.76
Patients' sex [male/female] (n)	11 / 9	13 / 9	χ^2^(*df* = 1) = 0.006, *p* = 0.938

After recruitment of tinnitus patients, a group of 22 healthy participants were included into the music therapy condition serving as active controls (AC). They were matched in age and sex to the patients' groups. They underwent the same therapy protocol as implemented in TG. This study protocol consisted in 9 consecutive 50-min sessions of individualized therapy over 5 days, comprising acoustic training for frequency discrimination, auditory attention control tasks, and guided exercises for mindfulness and distress regulation.

In total, data from 63 participants from the three groups were included in the analysis. The three samples used for Voxel Based Morphometry (VBM) did not differ in biometric data in sex [χ^2^(*df* = 2) = 0.99, *p* = 0.95] or in age profile [χ^2^(*df* = 2) = 1.76, *p* = 0.42].

### Study protocol

Therapy effects on tinnitus severity and individual tinnitus related distress were assessed by Tinnitus Questionnaire (TQ) developed by Goebel and Hiller (1998). The TQ refers to both tinnitus-related functional disabilities (such as concentration difficulties or hearing impairment) and emotional impairments (such as fear, anger or frustration due to tinnitus). TQ scores were obtained at three different times, during inclusion examinations, before start of treatment, and after the therapy week. The preceding TQ assessment as part of the inclusion examination was integrated into the experimental setup to exclude novelty effects from further evaluation of therapy effect.

All participants underwent two MRI sessions on two subsequent weekends. Between these MRI sessions, TG and AC were treated with music therapy according to the Heidelberg Model. Participants of PTC did not receive any intervention during this time. MRI scans were performed at the Department for Neuroradiology in Homburg using a “Skyra” Siemens 3-Tesla-Scanner and a 20-channel head coil. Each MRI session consisted of three parts: functional measurement during a continuous performance task previously used in attention studies (Schneider et al., 2010), high resolution anatomical T1-weighted scan, and functional measurement of emotional processing of tinnitus related (idiosyncratic) and other affective and neutral verbal stimuli (Golm et al., 2013). However, in this paper only the results from the anatomical scans will be reported. The Magnetization Prepaired Rapid Acquisition Gradient Echo (MPRAGE) protocol (Mugler and Brookeman, 1990) was used, resulting in a resolution of 0.9 × 0.9 × 0.9 isometric voxel size covering the whole head.

### Statistical analysis

MRI scans were performed twice, once before (A-image) and one after (B-image) the 1-week period, for the purpose of Voxel Based Morphometry (VBM) (Ashburner and Friston, 2000) as realized for longitudinal measurements by the VBM8-Toolbox (Christian Gaser, University of Jena, http://dbm.neuro.uni-jena.de/vbm). Brain compartments of white and gray matter were segmented, DARTEL normalized by IXI-template to MNI space (Ashburner, 2007), and smoothed by Gaussian kernel of 10 mm radius.

Comparisons of structural changes were calculated by “flexible factorial design” as implemented in SPM8 (Wellcome Trust Centre for Neuroimaging, London, 2010). The numerical procedure was carried out as a Two-Way ANOVA calculating the influence of the three participant groups and the two dependent time points, scanned before and after the study week, respectively. A comparison between treated and untreated patients (TG vs. PTC) was performed to examine differential therapy-induced effects on structural change. Resulting structural findings from this contrast were further used as spatial mask for general effects of music therapy. Specific tinnitus-related therapy effects were calculated by contrast between TG and “treated” AC in conjunction with selected brain clusters from general effects (TG vs. PTC). This step was implemented to separate tinnitus-related effects from general therapy-related effects. All obtained clusters of each comparison were corrected *post-hoc* by extent threshold of 125 contiguous voxels and reported after family-wise error (FWE) correction on cluster-level of 5% alpha error.

Revealed clusters from GM contrasts between groups were anatomically assigned to brain structures using the cytoarchitectonic maps as published in Morosan et al. (2001) by application of the Anatomy Toolbox (Eickhoff et al., 2006) supplemental to SPM.

### Music therapy and assessment of clinical therapy effects

The music therapy according to the Heidelberg Model of Music Therapy for tinnitus (Argstatter et al., 2008) is a manualized short term treatment approach lasting for nine consecutive 50-min sessions of individualized therapy. Therapy takes place over five consecutive days with two therapy sessions per day. The therapy was carried out by a team of two expert therapists, usually one music therapist and one psychotherapist.

Treatment by music therapy was characterized by several distinctive features:

1. Integration of both active as well as receptive techniques, not passive suppression of the tinnitus sound, but rather self-effective influence on the sounds.
2. Acoustic attention control by active participation, particularly in the form of vocal exercises both during and between music therapy sessions.
3. Improvement of acoustic perception by means of detailed training on intonation and listening capacity in the range of the transposed tinnitus frequency.
4. Musically based training in relaxation and well-being in order to decouple tinnitus from psychophysiological reaction patterns.
5. Tinnitus counseling using educational techniques focusing on individual tinnitus-related problems.

The treatment modules are described in more detail by Argstatter et al. (2012).

## Results

### Clinical therapy effect as assessed by TQ

**Figure 1:**
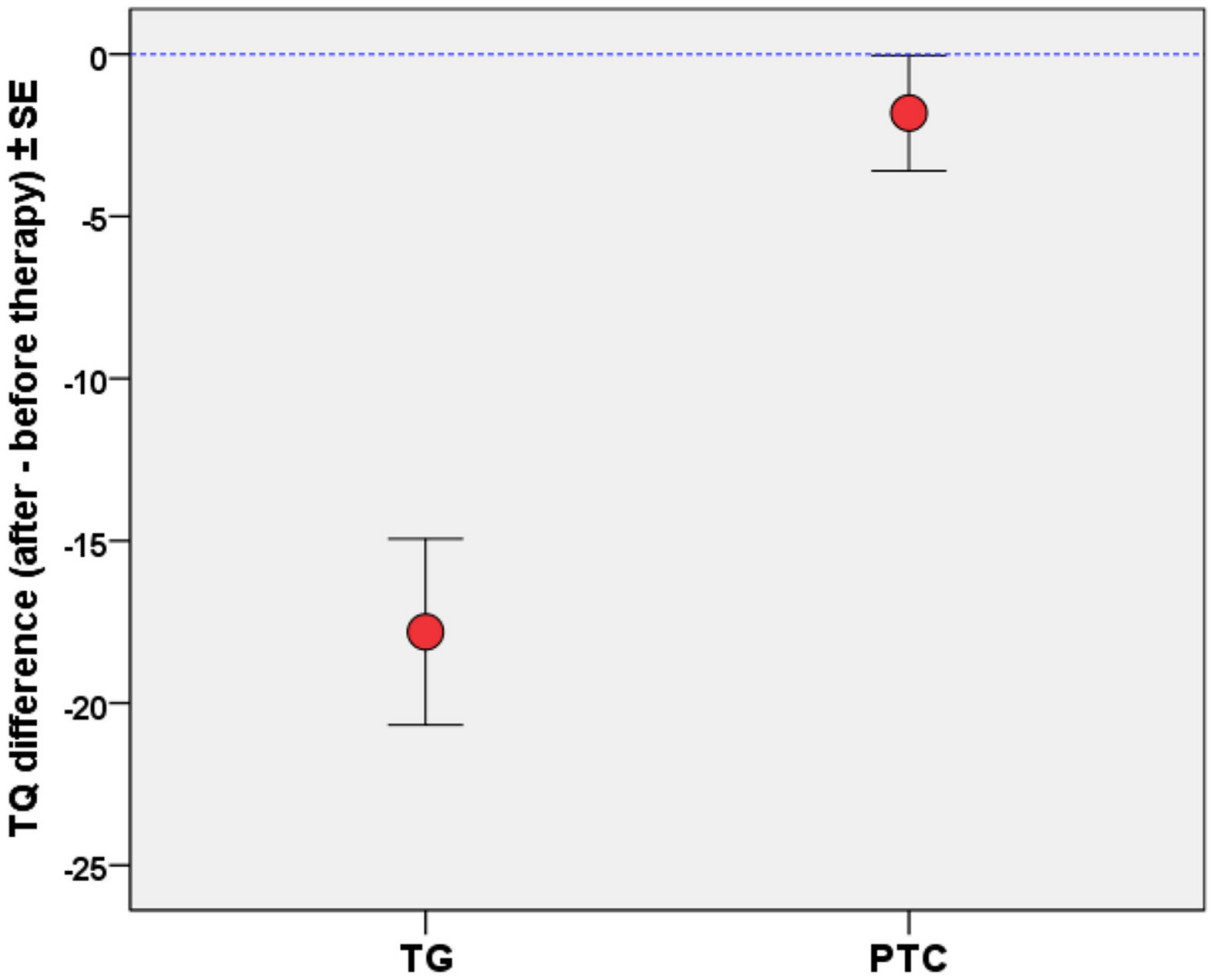
**Differences (after minus before) of Tinnitus Questionnaire (TQ) score**. Treatment by Heidelberg Model of Music Therapy led to decrease of 16 score points as compared to untreated patients on average (error bars: standard error of mean).

Tinnitus-related mental load in terms of distress or psychiatric disorders was measured by TQ at T_0_ (pre-participation evaluation), at T_1_ (therapy start) and therapy end (T_2_). The resulting therapy effect was assessed by the difference of TQ scores between T_1_ and T_2_. Treatment by the compact approach of the Heidelberg Model of Music Therapy over 1 week added up to 450 min of therapy sessions (9 × 50 min). TQ effects between TG and untreated PTC patients were assessed by General Linear Model (*df* = 1; *F* = 22.9; *MSE* = 1374) for repeated measures using SPSS21 (IBM Corp.). The 1-week therapy resulted in a significant (*p* < 0.00005) effect on change in TQ scores (see Figure 1) between both groups of tinnitus patients: Compared to a slight test-retest effect (about 1.8 TQ scale points) in PTC, in TG a significant (*T* = −5.7, *df* = 18, *p* < 0.0001) decrease of 17.7 (SD 13.6) TQ scale points was measured. In PTC, the TQ score did not significantly change over the observation period of 1 week.

### Therapy- related cortical alteration: GM increase in TG vs. PTC

**Figure 2:**
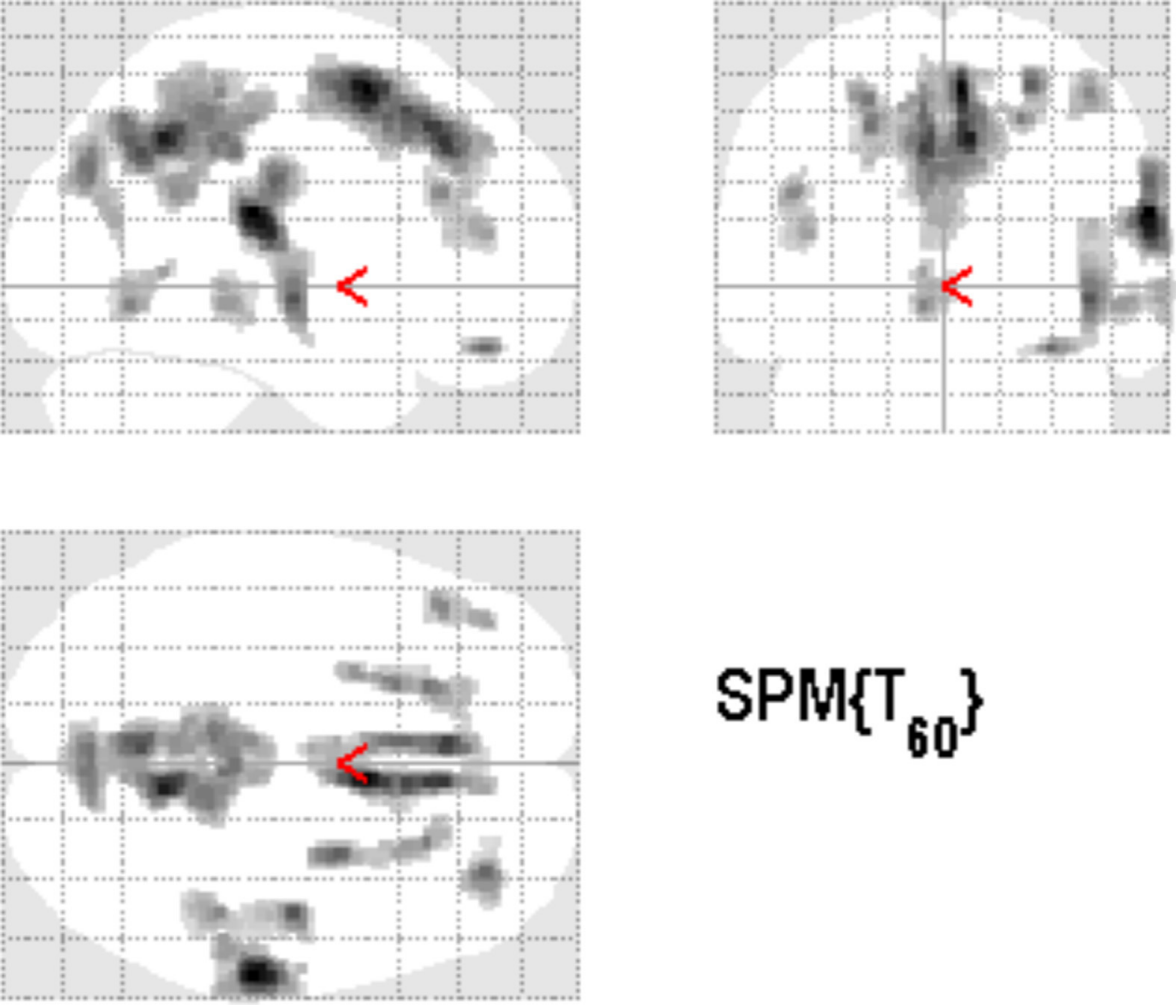
**General structural effects during the therapy week in comparable samples resulted in widely spread increased GM density in TG vs. PTC over 1 week (*p* < 0.001 uncorrected, 125 voxels extent threshold)**. Clusters in precuneus, medial superior frontal areas, and in the auditory cortex were found.

Over the observation period of 1 week, Heidelberg Model of music therapy was applied to TG patients, whereas PTC patients were not treated during this time span. Comparing structural alterations between T_1_ and T_2_, several brain regions revealed increased GM density in treated vs. untreated tinnitus patients (see Figure 2), yielding clusters in precuneus, supplemental motor area (SMA), medial superior frontal sulci, prefrontal areas, right Rolandic operculum and right Heschl gyrus (see Table 2). The highest effect was measured in the right Rolandic operculum resulting in GM increase of about 1.7% in treated patients. The resulting contrast was subsequently considered as a mask for general effects of the therapy situation, comprising training tasks, relaxation sessions, and specific auditory exercises.

**Table 2:** **Clusters showing increase of gray matter density in TG vs. PTC over 1 week**.

**Cluster**	**MNI (x y z)**			**p (cluster)**	**Size (vox.)**
**TG > PTC**					
Precuneus	8	−51	43	<0.001^**^	3417
SMA	5	9	57	<0.001^**^	1896
Right Rolandic Operculum/IPC/STG	62	−24	21	<0.001^*^	776
Right Heschl Gyrus	45	−16	7	0.003^*^	450
Left superior frontal sulcus	−21	27	46	0.006	356
Right superior frontal sulcus	26	0	58	0.015	268
Right middle temporal gyrus	56	−33	−3	0.042	176
Right IPC/postcentral	44	−34	56	0.044	172
Left IFG pars triangularis (BA45)	−44	32	27	0.049	165
Left cerebellum (lobus V)	−5	−61	−5	0.065	141
Right middle orbital gyrus	32	44	−17	0.074	132
**PTC > TG**					
None					

** p < 0.01/

* *p < 0.05 after FWE correction*.

### Cortical alterations in “treated” AC vs. untreated PTC

**Figure 3:**
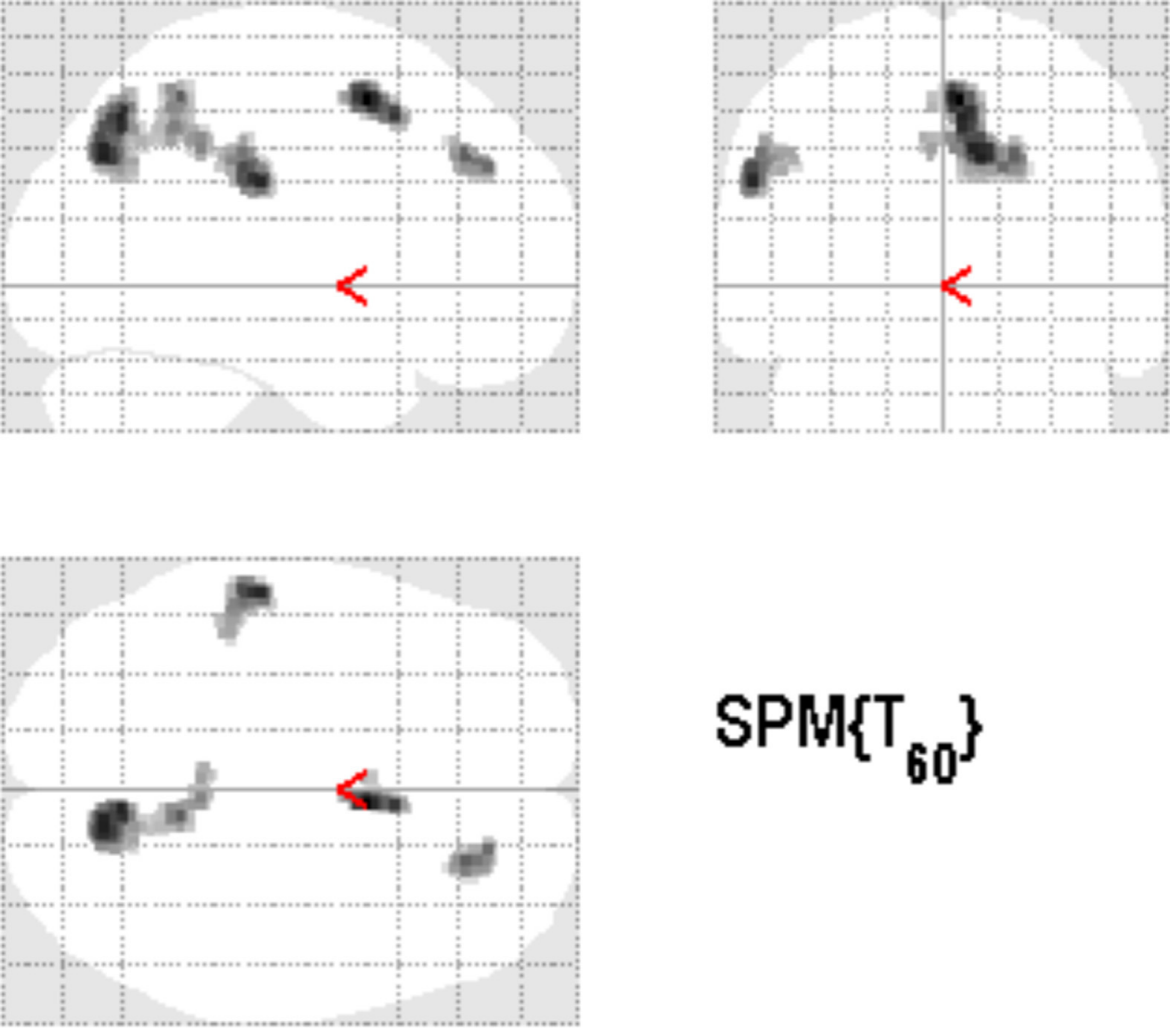
**Over the study week, clusters with increased GM density also were observed comparing AC vs. PTC in precuneus and frontal lobe (*p* < 0.001 uncorrected, 125 voxels extent threshold)**.

In “treated” AC, an increase of GM density also occurred in precuneus and medial superior frontal areas (see Figure 3) when contrasted with untreated PTC. This result overlapped with clusters revealed by contrast between TG and PTC. However, the contrast in AC did not reach the magnitude of the effect as observed in TG (see Table 3). No Clusters in temporal areas were observed.

**Table 3:** **Clusters representing increased gray matter density in AC vs. PTC over 1 week**.

**Cluster**	**MNI (x y z)**			**p (cluster)**	**Size (vox.)**
**AC > PTC**					
Right precuneus (BA7A)	11	−69	37	0.001^*^	562
Left IPC/postcentral	−56	−22	30	0.019	248
Right superior parietal lobe (BA5M)	8	−46	55	0.044	172
Left and right SMA (BA6)	5	9	54	0.053	158
Right superior frontal gyrus	21	39	37	0.081	125
**PTC > AC**					
None					

* *p < 0.05 after FWE correction*.

### Tinnitus-specific GM alterations: contrast between TG vs. AC

**Figure 4:**
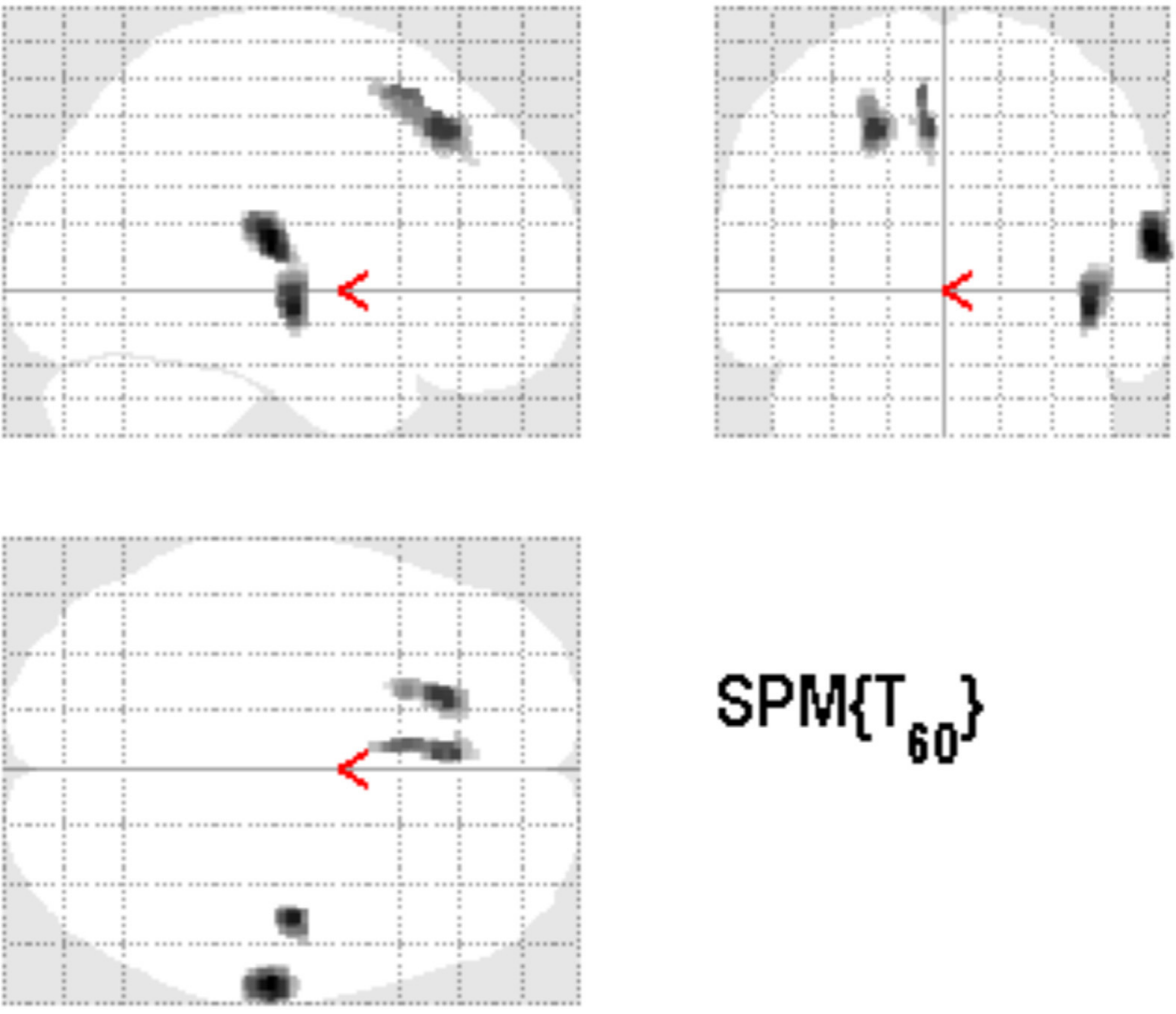
**Tinnitus-related alterations by comparison between TG and AC, each with experience of music therapy, were separated within the therapy-related alterations as measured by the contrast between TG and PTC (*p* < 0.001 uncorrected, 125 voxels extent threshold)**. This intersection of effects reveals clusters in right Heschl's gyrus, right Rolandic operculum, and medial superior frontal regions.

Comparison of TG and AC each with experience of the therapy week, respectively, but different in tinnitus presence, was performed to assess specific tinnitus-related structural effects due to the therapeutic intervention. This contrast was calculated within the clusters from general effects of the music therapy as calculated by contrast between TG and untreated PTC. Thus, this calculation is regarded as a selection of tinnitus-related effects within therapy-related structural GM alterations. Figure 4 shows the resulting clusters in right auditory and medial superior frontal areas, covering the right Heschl gyrus and the right Rolandic operculum (see Table 4).

**Table 4:** Separation of tinnitus-related from therapy-related GM alteration (^*^p < 0.05 after FWE correction) by calculating contrast TG > AC within mask of general therapy effect (TG > PTC).

**Cluster**	**MNI (x y z)**			**p (cluster)**	**Size (vox.)**
**(TG > AC) ∩ (TG > PTC)**					
Right Rolandic operculum (OP1)	63	−19	13	0.022*	233
Right Heschl Gyrus	50	−10	4	0.047*	167
Left superior frontal sulcus	−20	30	48	0.047*	167
Left medial superior gyrus	−5	17	58	0.064	143

## Discussion

The present study was very innovative due to the homogeneity of patient samples. Most of previous studies on neural correlates of tinnitus distress have been carried out on patients with chronic tinnitus in which tinnitus duration was not taken into account. Importantly, in this paper, these potentially moderating effects of tinnitus duration were explicitly controlled for by including only patients with a recent onset of tinnitus persisting for a maximum of 3 months. On the whole, only a few studies so far have engaged in systematic measurements of such neural alterations in *acute* tinnitus. Among them, Job et al. (2012) found neural hyperactivities in attention and emotion related areas especially in the insula, the ACC and the PFC in military adults with acute acoustic trauma and consequent tinnitus. In addition, Vanneste et al. (2011) examined the differences of the neural network between tinnitus of recent onset and chronic tinnitus. Their results indicate that the neural structures detected in both acute and chronic tinnitus were identical (comprising auditory cortices, insula, dorsal ACC and premotor cortex) but they also revealed different activity and connectivity patterns within this network.

In line with the previous findings of Argstatter et al. (2012) as well as of Grapp et al. (2013), a significant clinical improvement by the Heidelberg Model of music therapy was quantified using TQ. Thus, the neuro-music therapy approach according to the “Heidelberg Model” seems to provide an effective treatment option for patients with acute tinnitus if initial medical treatment fails to induce remediation. In these studies, both a significant improvement in subjectively perceived tinnitus distress and GM changes were evident immediately after the treatment. The improvements in tinnitus distress not only concerned the patients' cognitive and emotional strategies dealing with tinnitus, but also its intrusiveness and subsequent auditory perception difficulties. Compared to most other therapy options for tinnitus patients, the Heidelberg Model of Music Therapy goes far beyond a pure symptom management. At the core of this treatment approach, the patients are “confronted” actively with their individual tinnitus sounds and are instructed to deal with their tinnitus explicitly instead of trying to ignore them.

In the present study, results revealed that, consistent with the clinical effects of music therapy, GM increased substantially in treated patients (TG) as well as in active controls (AC) compared to untreated patients (PTC). Both TG and AC experienced the same exercises and therapeutic sessions. However, GM increase in treated patients covered more brain areas and yielded higher effect sizes compared to the AC. One may speculate on these findings that healthy controls did not similarly profit from the therapeutic interventions as did tinnitus patients. While tinnitus distress is treated both by relaxation techniques and by frequency discrimination exercises, healthy subjects probably experienced these approaches as “mental wellness” only due to their general influence on the distress network (De Ridder et al., 2013). This may contribute to explain the different effect sizes despite equal training schedule. However, this difference was expected due to a specificity of therapeutic effects on patients suffering from tinnitus (compensation view).

The tinnitus-related structural effects, or the therapy-induced GM alterations, respectively, could be consistently located on areas that are considered to be most sensitive for tinnitus-related distress (Leaver et al., 2012; De Ridder et al., 2013; Schecklmann et al., 2013). However, findings on the direction of the structural therapy effects revealed in our study were not in line with previous findings on tinnitus distress: Whereas mental tinnitus load had been previously associated with GM loss in Heschl's gyri and in dorsomedial frontal location, improvement by music therapy intervention resulted instead in GM increase in these areas.

Most probably music therapy was able to influence and reinforce auditory sensation of those frequencies that were disrupted by a partial hearing impairment. Many patients reported a variability of tinnitus pitch in the course of therapy sessions and lower tinnitus loudness after the therapy week (Hutter et al., 2014). As this partial hearing loss is considered to cause the phantom noise (Lanting et al., 2009), exercises of frequency discrimination in the spectral range of tinnitus might be involved in the reduction of distress and loudness. Although a rapid direct cochlear regeneration can be deemed implausible, further compensation strategies using overtone or envelope characteristics of musical harmonics can be trained to enhance signal extraction for auditory processing. A higher perceptual efficiency regarding the defective frequencies is then able to more activate those areas within auditory cortex that formerly sustained a loss of GM due to lack of signal. More neuronal activation in turn modulates reconstruction processes in the neuronal network, for example, by a down regulation of restrictive factors for neuronal contact in the peri-neuronal net (Wang and Fawcett, 2012). Although VBM is not able to directly depict cellular activity, one may assume that these regeneration processes are similar to re-innervation mechanisms, involving the peri-neuronal net proteins (Kwok et al., 2011). Results from training studies in a mouse model indicate a subsequent growth of synaptic bulk accompanied by a dilatation of the neuronal network on tissue level over several days (Kleim et al., 2004). On a macroscopic level, this tissue augmentation can be detected as a rapid increase of GM density by structural MRI (Warraich and Kleim, 2010). Driemeyer et al. (2008) also underlined these temporal dynamics of structural plasticity by training in humans. They observed a major increase of GM as early as after 7 days during a continuous motor coordination training task. Recent studies even report a reduction of training-induced cortical reorganization despite ongoing exercises over longer observation times (Tennant et al., 2012). This also may explain our results of striking GM alterations due to the compact therapy over 1 week.

In contrast to bilateral results of Schecklmann et al. (2013), the music therapy influenced the right auditory areas only at the reported statistical level. This lateralization may be based on functional lateralization in auditory processing (Warrier et al., 2009), indicating that the right Heschl's gyrus might be more involved in spectral-related acoustic information. In general, the left primary auditory cortex is more active in right-handed subjects, but it shows more sensitivity to temporal stimulus variation compared to frequency variation (Izumi et al., 2011). However, a frequency discrimination task requires more involvement from the right auditory cortex (Doeller et al., 2003). Thus, the Heidelberg Model of music therapy comprising exercises of frequency discrimination in the impaired spectral range was able to specifically repair the tinnitus-related GM loss in the right Heschl's gyrus.

### Limitations

Limitations of the study should be discussed. The TQ scores of included tinnitus patients ranged from 7 to 67 with an average score of 37.3 ± 16. This value corresponds to mild or middle tinnitus-related distress. Further, only patients with general hearing impairment less than 40 dB were included for the present. Therefore, therapy success in severe cases cannot be predicted by this study.

Another limitation generally concerns the measurement of gray matter alterations by SPM and VBM. Due to usage of non-linear deformation, there is some residual impreciseness during the overlap of gyri and sulci between individual brains. Although we calculated with repeated intra-subject measures, the assignment of individual contrasts to the standard space must be critically regarded. The relative quality of the DARTEL normalization used in the study has been compared with several other methods by Klein et al. (2009), resulting in an acceptable rating.

Further limitations are related to possible interpretations of VBM contrasts indicating a shifted probability of focal GM or WM proportion. It is hard to decide whether its origin may be found in some growth within certain brain structures or slightly shifted segmentation results due to certain tissue alteration.

## Conclusion

The Heidelberg Model of Music Therapy was able to reveal both rapid clinical improvements related to tinnitus distress and evidence of this specific therapeutic effect on brain areas suspected to play a role in sustaining tinnitus-related distress. When taking into account that the Heidelberg Model of Music Therapy has been shown to provide long-lasting effects (Argstatter et al., 2012), the observed structural brain plasticity can be assumed to be causative. Due to the rapid intervention in acute tinnitus this therapy may be able to prevent tinnitus from chronification (Grapp et al., 2013).

### Conflict of interest statement

The authors declare that the research was conducted in the absence of any commercial or financial relationships that could be construed as a potential conflict of interest.
